# Designing a novel mRNA vaccine against *Vibrio harveyi* infection in fish: an immunoinformatics approach

**DOI:** 10.5808/gi.21065

**Published:** 2022-03-31

**Authors:** Sk Injamamul Islam, Moslema Jahan Mou, Saloa Sanjida, Muhammad Tariq, Saad Nasir, Sarower Mahfuj

**Affiliations:** 1 Department of Fisheries and Marine Bioscience, Faculty of Biological Science, Jashore University of Science and Technology, Jashore 7408, Bangladesh; 2 Chulalongkorn University, Department of Veterinary Microbiology, Faculty of Veterinary Science and Technology, Bangkok 10330, Thailand; 3 Department of Genetic Engineering & Biotechnology, Faculty of Earth and Life Science, University of Rajshahi, Rajshahi 6205, Bangladesh; 4 Department of Environmental Science and Technology, Faculty of Applied Science and Technology, Jashore University of Science and Technology, Jashore 7408, Bangladesh; 5 Department of Biotechnology, Faculty of Biological Sciences, University of Malakand, Chakdara 18800, Pakistan; 6 Department of Clinical Medicine and Surgery, Faculty of Veterinary Medicine, University of Veterinary and Animal Sciences, Lahore 54000, Pakistan

**Keywords:** immune simulation, molecular dynamics simulation, T-cell epitopes, vaccine design, *Vibrio harveyi*

## Abstract

*Vibrio harveyi* belongs to the family Vibrionaceae of class Gammaproteobacteria. Around 12 Vibrio species can cause gastroenteritis (gastrointestinal illness) in humans. A large number of bacterial particles can be found in the infected cells, which may cause death. Despite these devastating complications, there is still no cure or vaccine for the bacteria. As a result, we used an immunoinformatics approach to develop a multi-epitope vaccine against the most pathogenic hemolysin gene of *V. harveyi*. The immunodominant T- and B-cell epitopes were identified using the hemolysin protein. We developed a vaccine employing three possible epitopes: cytotoxic T-lymphocytes, helper T-lymphocytes, and linear B-lymphocyte epitopes, after thorough testing. The vaccine was developed to be antigenic, immunogenic, and non-allergenic, as well as have a better solubility. Molecular dynamics simulation revealed significant structural stiffness and binding stability. In addition, the immunological simulation generated by computers revealed that the vaccination might elicit immune reactions *Escherichia coli* K12 as a model, codon optimization yielded ideal GC content and a higher codon adaptation index value, which was then included in the cloning vector pET2+ (a). Altogether, our experiment implies that the proposed peptide vaccine might be a good option for vibriosis prophylaxis.

## Introduction

The disease, called vibriosis, affects both farmed and marine fish across the globe. The pathogenicity of *Vibrio* species and their antibiotic resistance is, however, poorly understood. The virulence components of *Vibrio* spp. that have been associated to animal and human illnesses are often not accessible or common in the environment [1]. Because *Vibrio* has a highly plastic genome, there is a substantial likelihood that pathogenic and ambient *Vibrio* will share genes for virulence. As a result, there has been an increase in the number of pathogenic *Vibrio* strains in the aquatic environment [2]. *Vibrio harveyi* is one of the most serious infections affecting farmed fishes, and *Vibrio alginolyticus*, *Vibrio parahaemolyticus*, and *Vibrio campbellii* have also been found in numerous tropical nations [3-6]. Among temperate waters of Asia, southern Europe, and South America, this pathogen occurs naturally in marine habitats and has become an important pathogen of wild and cultured fish and invertebrates. The symptoms of *V. harveyi* infection include anemia, necrosis of the intestine, ascetic fluid, petechial hemorrhages, tail erosion, infection of the eye, mucous secretions, and frequent mortality in fish [7]. Moreover, resistance to bactericidal processes is one of the most important aspects in the pathogenicity of fish infections. Overuse of antibiotics in human medicine, agriculture, and aquaculture systems has resulted in the emergence and evolution of antimicrobial resistance in *Vibrio* spp. over the last few decades [8]. *V. harveyi* is highly pathogenic to salmonids, sea bass, and tilapia, and generates an extracellular product with a high titer of hemolytic activity against fish erythrocytes. *V. harveyi* has a single chromosome with a length of 6,374,398 base pairs [9]. In spite of several genes associated with *V. harveyi* causing disease in fish, hemolysin is well-known as a virulence factor linked to both fish and human diseases [10]. The hemolysin protein is a crucial protein that allows viruses to penetrate the host cell wall, making it a suitable target antigen for vaccine development [11]. Cytotoxic T lymphocyte (CTL) epitopes [12] and CD4+ T cell epitopes were found to diverge in fish species [13] by epitopes mapping using fish with experimentally infected disease and a library of overlapping peptides of viruses [14]. Immunizations are intended to elicit an immune response to a potentially lethal foreign pathogen and to prepare the body to infiltrate those particles, limit toxicity, or initiate assassination activities against the bacteria. A vaccination, according to prior research, can prevent future outbreaks of bacteria-associated natural microorganisms such as bacteria [15]. The prompt discovery of safe, efficient, uncomplicated, economical, dependable, and fast production of antibody against the guided antigen is made possible by *in-silico* design of multi-epitope vaccines against pathogens. Epitope-based vaccines have been successfully created in the postgenomic period to stimulate responsiveness against some of the worst human viruses, including influenza, nipah, chikunguniya, zika, ebola, Middle East respiratory syndrome coronavirus, rota, and others [16-20]. Previously, the *in-silico* technique in fish had not been developed due to a lack of understanding of the differences between major histocompatibility complexes (MHC class I and II) and human leukocyte antigen (HLA) [21,22] but recent research on fish species has generated data to enable *in-silico* techniques [23-25]. Both MHC class I and class II molecules were found in the experimental data of cord and tilapia for starting immune responses against infections. As a result, the peptide with excellent binding capacities to HLA-A*0201, HLA-B*3501, and HLA-B*3508 might be employed as efficient vaccinations against certain fish diseases [21,26]. Lately, an *in-silico* technique was effective in predicting epitopes and multiepitopes with significant responsiveness against *Streptococcus agalactiae, Edwardsiella tarda*, and *Flavobacterium columnarie*, three harmful bacteria that induce streptococcosis, edwardsiellosis, and columnaris in fish, separately [27-29]. Experts expect that in the coming days, computer-assisted techniques will be increasingly successful in controlling fish diseases [30,31]. As a result, the main objective of this research was to identify multi-epitope from the best antigenic protein to fight against *V. harveyi* infection.

## Methods

**Fig. 1.**
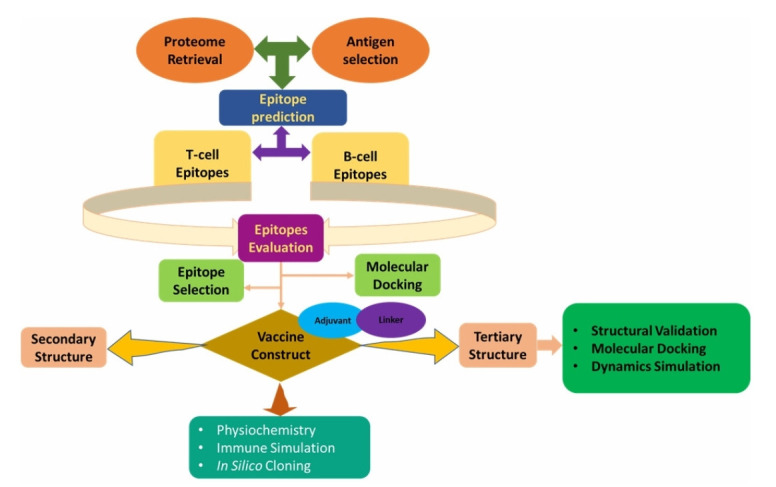
Architectural flow chart of the study.

Architectural flow chart is being given in Fig. 1.

### Retrieval of proteome and antigen selection

We used the NCBI (https://www.ncbi.nlm.nih.gov/) database to find accessible *Vibrio harveyi* proteomes for antigen selection. Hemolysin is a crucial protein that allows viruses to penetrate the host cell wall, making it a suitable target antigen for *V. harveyi* vaccine development [32,33]. The hemolysin is a vibriosis component in fish that is thought to be responsible for causing mortality in fish [34]. We examined the hemolysin protein of the *V. harveyi* for multi-epitope vaccine design because of its direct role in pathogenesis. After the hemolysin was isolated, the chosen amino acid sequences of the bacteria were obtained as FASTA files (GenBank: ACF32997.1). VaxiJen v2.0 (http://www.ddg-pharmfac.net/vaxijen/) server was used to assess the protective antigens of hemolysin [35] and for each of them, a threshold value of 0.4 was chosen on the ANTIGENpro (http://scratch.proteomics.ics.uci.edu/) server [36]. Subsequently, the hemolysin with the highest antigenic score was chosen for further research.

### Prediction and assessment of cytotoxic T-lymphocyte epitope

CTLs are basic kinds of immune responsive cells that have the ability to directly destroy other infectious cells [37]. They immediately enter the infected cell and contribute to the host's defensive response. The sequence of the chosen protein was entered into a server named NetCTL v1.2 (http://www.cbs.dtu.dk/services/NetCTL/) to predict CTLs epitope [38]. It integrates information about proteasomal C terminal cleavage affinity (C-score), TAP transport efficiency, and MHC class I affinity to deliver its output for a given protein. The threshold parameter for prediction was set to 0.4 to obtain 0.89 sensitivity and 0.940 specificity. VaxiJen v2.0 was used to further evaluate the predicted epitopes [35], followed by MHC class I immunogenicity (http://tools.iedb.org/immunogenicity/) [39], ToxinPred (http://crdd.osdd.net/raghava/toxinpred/) [40], and AllerTop v2.0 (https://ddg-pharmfac.net/OP/) [41] online servers. All of the forecasts were made using the default parameters of each server.

### Epitopes of helper T-lymphocytes prediction and assessment

In response to external antigens, helper T-lymphocytes (HTLs) detect and activate B lymphocyte and CTL and causing the infectious pathogen to be destroyed [42]. The HTL epitopes were defined using the IEDB's MHC class II binding allele prediction tool, which can be found at http://tools.iedb.org/mhcii/. With a percentile rank of 5%, the HTL epitopes were chosen using the CONSENSUS technique [43]. The IEDB currently recommends making selections based on a percentile rank of ≤1% for each (MHC allele, length) combination to cover most of the immune responses. Alternatively, a binding affinity (IC50) threshold of 500 nM identifies peptide binders recognized by T cells and this threshold can be used to select peptides [44]. This tool employs different methods to predict MHC class II epitopes, including a consensus approach which combines NN-align, SMM-align, and combinatorial library methods. The antigenicity and cytokine-inducing properties of the anticipated epitopes, namely interferon-γ (IFN-γ), interleukin-4 (IL-4), and interleukin-10 (IL-10), were assessed further. Antigenicity was predicted using the VaxiJen v2.0 server, while IFN-γ, IL-4, and IL-10 features were anticipated employing IFNepitope (http://crdd.osdd.net/raghava/ifnepitope/) [45], IL4pred (http://crdd.osdd.net/raghava/il4pred/) [45], and IL10pred (http://crdd.osdd.net/raghava/IL-10pred/) [46] servers, respectively, with default parameters.

### Prediction and assessment of linear B-lymphocyte epitopes

To promote humoral or antibody-mediated immunity, B-cell epitopes are required. B-cells are made up of amino acid groups that bind with secreted antibodies and stimulate the immune system to fight infections [47]. As a consequence, we utilized the iBCE-EL server (http://www.thegleelab.org/iBCE-EL/) to identify the linear B-lymphocyte (LBL) epitopes using default settings [48]. It is an ensemble method that combined extremely randomized tree and gradient boosting algorithms, which respectively utilizes a combination of amino acid composition and physicochemical properties and a combination of dipeptide and physicochemical properties as an input feature. For a given peptide, iBCE-EL predicts its calss and probability values [48]. This server also can give 12‒25 mer sequence as output. The VaxiJen v2.0, ToxinPred, and AllerTop v2.0 servers were used to test the anticipated LBL epitopes.

### Peptide modeling and molecular docking

PEP-FOLD v3.0 (https://bioserv.rpbs.univ-parisdiderot.fr/services/PEP-FOLD3/) server was used to simulate the chosen CTL and HTL epitopes. For the procedure, the sOPEP sorting scheme with 200 simulations was employed [49]. HLA-B*3508, HLA-A*0201, and HLA-B*3501were chosen for selected CTL epitopes, whereas DRB1*07:01, DRB1*04:01, and DRB1*11:01were chosen for HTL epitopes, based on epitope-wise HLA binding allele analysis. The HLA allele crystal structures were obtained from the Protein Data Bank (PDB) (https://www.rcsb.org/) [50] followed by processing with BIOVIA Discovery Studio 2017. The AutoDock program was used to construct a grid-box around the active site of each HLA allele for molecular docking. Furthermore, the AutoDock Vina script was used to perform molecular docking between the epitopes and their associated HLA alleles [51]. To compare epitope binding effectiveness, the corresponding co-crystal ligands were utilized as a positive control. BIOVIA Discovery Studio 2017 and PBDSum were used to visualize the docked complex.

### Formulating of multi-epitope vaccine

The vaccine was created by combining the chosen CTL, HTL, and LBL epitopes with a suitable adjuvant and linking them with the proper linkers [52,53]. Because Toll-like receptor 4 (TLR4) is recognized by viral glycoproteins, and the adjuvant is essential for optimum translation and synthesis of the target vaccine candidate, we employed TLR4 agonist as the adjuvant [54,55]. As a result, the adjuvant 50S ribosomal protein L7/L12 (NCBI ID: P9WHE3) was evaluated to boost the vaccine candidate's immunogenicity. With the EAAAK bi-functional linker, which can break apart two b domains with weakly interacting interactions over a wide range of peptide lengths, the adjuvant was attached to the vaccination front. In contrast, the selected CTL was linked with the help of Ala-Ala-Tyr (AAY) linkers, the HTL was linked with GlyPro-Gly-Pro-Gly (GPGPG) linkers and the LBL was linked with Lys-Lys (KK) linker [47,52]. The AAY linker is a proteasome cleavage site that has been exploited to modify protein stability, decrease immunogenicity, and improve epitope presentation [56]. With GPGPG, a 'junctional epitope' is avoided, which simplifies immune processing, while the bi-lysine KK linker helps to maintain the separate immunogenic properties of the vaccine construct.

### Physicochemical and immunological evaluation

The physiochemistry of a protein describes its fundamental characteristics. The ProtParam server, which can be found at https://web.expasy.org/protparam/, was used to predict the vaccine's physicochemical properties to comprehend the vaccine's essential essence [57]. We also evaluated the immunological properties through VaxiJen v2.0 [35], MHC-I immunogenicity [39], AllerTop [41], and SOLpro [36] servers.

### Secondary structure prediction

The two-dimensional (2D) structural features such as alpha-helix, beta-turn, and random coils of the construct were identified by SOPMA (Self-Optimized Prediction Method with Alignment) server at https://npsa-prabi.ibcp.fr/NPSA/npsa_seccons.html [58] and PSIPRED v4.0 (PSI-blast based secondary structure prediction) server at http://bioinf.cs.ucl.ac.uk/psipred/ [59] with default parameters. SOPMA has a prediction accuracy of above 80% [58]. To further understand the vaccine's composition quality, 2D structural characteristics were retrieved and assessed.

### Homology modeling, 3D structure refinement, and validation

The constructed vaccine was submitted into the RaptorX server (http://raptorx.uchicago.edu/) [60]. Using a cutting-edge algorithm and a 3D structure, the RaptorX server produces the most precise structure of the protein and its activities [60]. The C-score, TM-score value, root mean square deviation (RMSD), and top five models of a particular protein sequence may all be predicted and determined using this web service. The generated 3D structure was saved as a PDB file, which was chosen based on the C-score. The C-score on the server ranges from –5 to 2, with a higher number indicating a more confident protein model. For the refining of the vaccine structure, the discovered 3D structure was uploaded to the GalaxyRefine (http://galaxy.seoklab.org/refine) online web-based server. The CASP10 refining approach was used to operate this webserver [61]. The RMSD, energy score, and overall quality score are all available on the GalaxyRefine website. The improved structure was downloaded, and the chosen structure was determined using the energy scores of the lowest and maximum RMSD values. PyMOL v2.3.4 was used to show the refined and discovered structure [62]. The Ramachandran plot score (vaccine structure validity) and Z-score value, which identify the standard deviations from the mean value, were used to analyze the final 3D structure. The Ramachandran plot was analyzed by the Rampage server (http://mordred.bioc.cam.ac.uk/rapper/rampage.php), which runs considering allowed and disallowed regions of amino acid [63]; and Z-score plot was analyzed by the ProSA-web (https://prosa.services.came.sbg.ac.at/prosa.php) tool [64].

### Molecular docking studies

The binding interactions between modeled proteins and receptor molecules can be revealed through molecular docking experiments. For this, we used the ClusPro v2.0 server, which can be found at https://cluspro.bu.edu/, to submit the refined vaccine model as a ligand and the TLR4 protein as an immunological receptor for molecular docking [65]. The TLR4 receptor was chosen and downloaded from the PDB server (PDB ID: 4G8A). Separating the associated ligand from the protein was the first step in preparing the receptor, which was followed by the removal of water and other chemicals. All of these procedures were carried out using the PyMOL v2.3.4 program [62]. Discovery Studio 2017 and PBDSum were used to investigate binding interactions and residues in the interacting surface.

### Molecular dynamics simulation

The complex structure of the selected candidate compounds was evaluated using 50 ns molecular dynamic simulations (MDS) to evaluate their binding stability to the desired protein to the active site cavity of the protein [66]. The MDS of the receptor-ligand complex was performed using the ‘Desmond v6.3 Program' in Schrödinger 2020-3 under Linux framework to evaluate the thermodynamic stability of the receptor-ligand complex [67]. To solve the system, a predetermined TIP3P water model was used, with an orthorhombic periodic boundary box form with a box distance of 10 Å assigned to both sides to retain a specific volume. After constructing the solvated system containing protein in complex with the ligand, the system has been minimized and relaxed using the default protocol introduced within the Desmond module with OPLS_2005 force field parameters [67]. In protein preparation wizard: Initially, protein preprocesses by adding hydrogens, create disulfide bonds, fill in the missing side chains, and delete waters using Epik (pH: 7.0 ± 2.0) and optimize by PROPKA pH: 7.0. In model system for simulation run, simulation time = 50 ns, trajectory intervals = 50 ps, total number of frames = 1,000, Ensemble class = NPT, temperature = 300 K, and one atmospheric (1.01325 bar) pressure. Finally, the simulation was carried out for 100 ns, and root mean square fluctuation (RMSF), RMSD, and protein secondary structure elements from the trajectories were analyzed to reveal the stability of the vaccine complex.

### Immune response simulation

Using the C-IMMSIM v10.1 server (http://www.cbs.dtu.dk/services/C-ImmSim-10.1/), the entire construct was uploaded for assessment of the vaccine's potential immunological response [68]. As previously stated, we used a minimum gap of 30 days between two dosages in this situation [69]. Three injections were administered in silico with time steps of 1, 84, and 168, respectively, where one-time step equals 8 h in real life. With the maximum simulation step value set to 300, all other stimulation parameters were left at their default settings.

### Codon adaptation and *in-silico* cloning

Codon optimization is required for the expression of a foreign gene in a host organism [70]. As a result, the construct was uploaded to the JCat service for codon adaptation (http://jcat.de/). We employed the commonly used *E. coli* K12 as the host in this study, and the entire procedure was carried out while avoiding the following three criteria: Sites of restriction enzyme cleavage, binding sites of prokaryotic ribosomes, and rho-independent transcription termination. The codon adaptation index (CAI) value and guanine–cytosine (GC) concentration of the modified sequence were used to evaluate it [70]. Lastly, the *in-silico* cloning of the adapted nucleotide sequence into the pET28a (+) expression vector was performed using the modified nucleotide sequence. SnapGene v4.2 software was used to carry out the entire *in-silico* cloning procedure [71].

## Results

### Highest antigenic protein selection

The retrieved *V. harveyi* proteomes featured hemolysin protein. We chose a hemolysin protein with the highest antigenic score of 0.4070 (VaxiJen) and 0.617 (ANTIGENpro) from all examined proteins based on antigenicity. The chosen hemolysin had a length of 418 amino acids and a GenBank accession number of ACF32997.1. For subsequent investigation, the main sequence of the chosen protein was employed.

### Potential CTL epitopes

To design a rational vaccine, accurate predictions of CTL epitopes are crucial. Furthermore, they can minimize the amount of experimental effort needed to identify epitopes. From the chosen hemolysin protein, a total of 52 CTL epitopes with a length of nine amino acids were predicted by using NetCTL v1.2 server. 22 CTL epitopes were shown to be antigenic, immunogenic, non-toxic, and non-allergenic. We chose the top three CTL epitopes for the final vaccine design based on the antigenicity score due to the large number of possible epitopes (Table 1). C-score is the combined score provided by the NetCTL server.

### Potential HTL epitopes

Initially, the IEDB server was used to identify 358 HTL epitopes, each with a length of 15 amino acids. Only 14 HTL epitopes were able to trigger the three kinds of cytokines tested, including IFN-γ, IL-4, and IL-10. Similarly, based on the antigenic score, we examined the top three HTL epitopes for incorporation into the final vaccine design (Table 2).

### Potential LBL epitopes

To develop epitope-based vaccines, produce antibodies, and prevent and diagnose diseases, B-cell epitopes must be identified. In this study, a preliminary investigation found 10 LBL epitopes, each of which is 12 amino acids long. Later with further evaluation, two epitopes were found as antigenic, non-toxic non-allergenic (Table 3).

### Docking studies of epitope and alleles

**Fig. 2.**
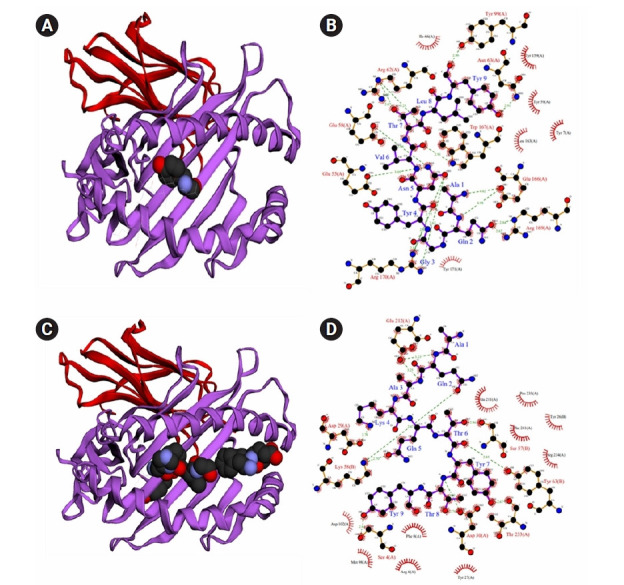
Interaction between epitopes and their respective binding alleles. As a representation of all-selected epitopes, we offer the docking interactions of the best HTL and CTL epitopes, where interaction between the HLA-B*3501 alleles and CTL epitope AQAKQTYTY (A, B) and docking between the DRB1*11:01 alleles and HTL epitope DATRAPQFTYSTQEE (C, D). HTL, helper T-lymphocyte; CTL, cytotoxic T lymphocyte.

The docking approach was utilized to confirm the efficiency of chosen epitopes in binding their HLA alleles. Table 4 lists the epitopes, as well as their corresponding docking alleles, binding affinities, interactions, and hydrogen-bonding residues. CTL epitopes had binding affinities of between –6.1 and –8.4 kcal/mol, while HTL epitopes had binding affinities of between –5.9 and –6.8 kcal/mol. In addition to the tabulated details, we presented the best interacting CTL (AQAKQTYTY) and HTL (DATRAPQFTYSTQEE) epitopes in Fig. 2. Herein, the best CTL epitope produced a total of nine hydrogen bonds, in which eight were classical interactions involved with the active site residue Tyr9, Leu8, Thr7, Glu166, Lys66, Arg170, Tyr4, Trp167, and Ala1. On the other hand, the best HTL epitope showed nine hydrogen bonds, including seven classical interactions while it interacted with Asp29, Lys58, Thr8, Asp30, Thr233, Ser57, Gln5, Glu212, and Lys4 residues.

### Vaccine construct and basic properties

**Fig. 3.**
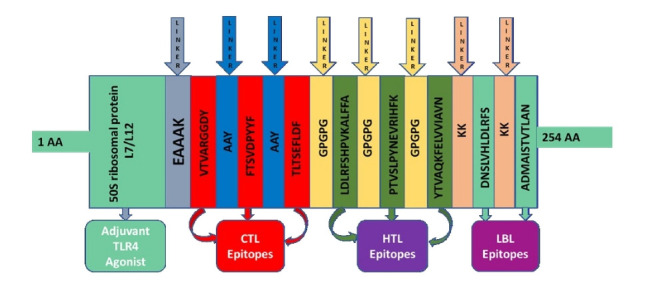
Graphical map of the formulated multi-epitope vaccine construct. Herein, the adjuvant and the first CTL epitope were linked by EAAAK linker, CTL epitopes were added together by AYY linkers, HTL epitopes by GPGPG linkers and LBL epitopes by KK linkers. CTL, cytotoxic T lymphocyte; HTL, helper T-lymphocyte; LBL, linear B-lymphocyte; TLR4, Toll-like receptor 4.

**Fig. 4.**
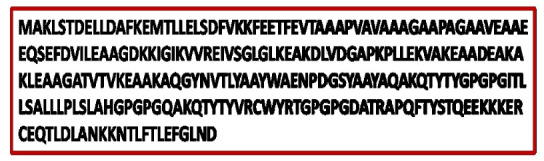
Constructed vaccine sequence.

The vaccine was created utilizing eight epitopes from three distinct classes that had previously been chosen (3 CTL, 3 HTL, and 2 LBL). As illustrated in Fig. 3, the epitopes were linked together using AAY, GPGPG, and KK linkers, respectively. To enhance immunogenicity, an adjuvant was applied before the construct. Using the EAAAK linker, the TLR4 agonist 50S ribosomal protein L7/L12 was connected to the initial CTL epitope as an adjuvant. The final vaccination had a length of 268 amino acids (Fig. 4).

### Physicochemical properties and immunological evaluation

**Fig. 5. f5-gi-21065:**
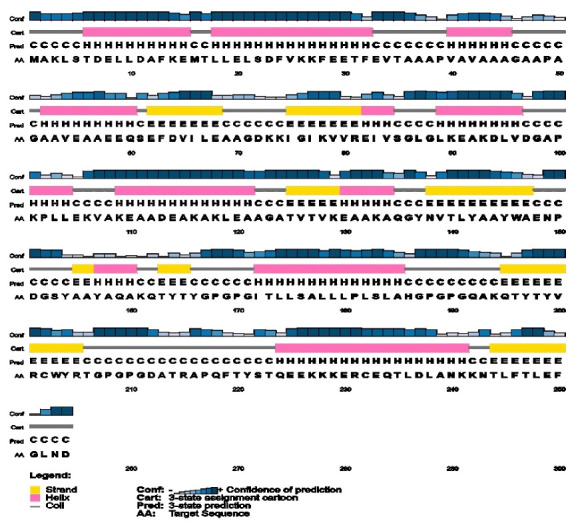
Secondary structure prediction of designed multitope vaccine using PESIPRED server.

Table 5 shows the physicochemical parameters of the vaccine construct. The construct was discovered to have a molecular weight of 27,044.64 Da. Other features such as the theoretical isoelectric point (pI) of 4.95, the chemical formula of C_1214_H_1914_N_310_O_379__S4_, the instability index of 20.25, the aliphatic index of 82.05, and the grand average of hydropathicity of ‒0.237 were also present. The construct's physicochemical properties and immunological efficacy were also assessed. For example, the construct's antigenicity was 0.7017, whereas its immunogenicity was 1.59238. Furthermore, the vaccine was non-allergenic and soluble, with a score of 0.891723 out of 1 (Table 5). α-helix, β-strand, and random coils were examined utilizing two distinct servers as secondary structural characteristics. The SOPMA server predicted 33.07% α-helix, 16.93% β-strand, and 50% random coils in the construct (Table 6). On the other hand, the PSIPRED server anticipated the features as 42.91% a-helix, 20.47% b-strand, and 36.61% random coils (Table 6, Fig. 5).

### Tertiary structure, refinement, and validation

**Fig. 6.**
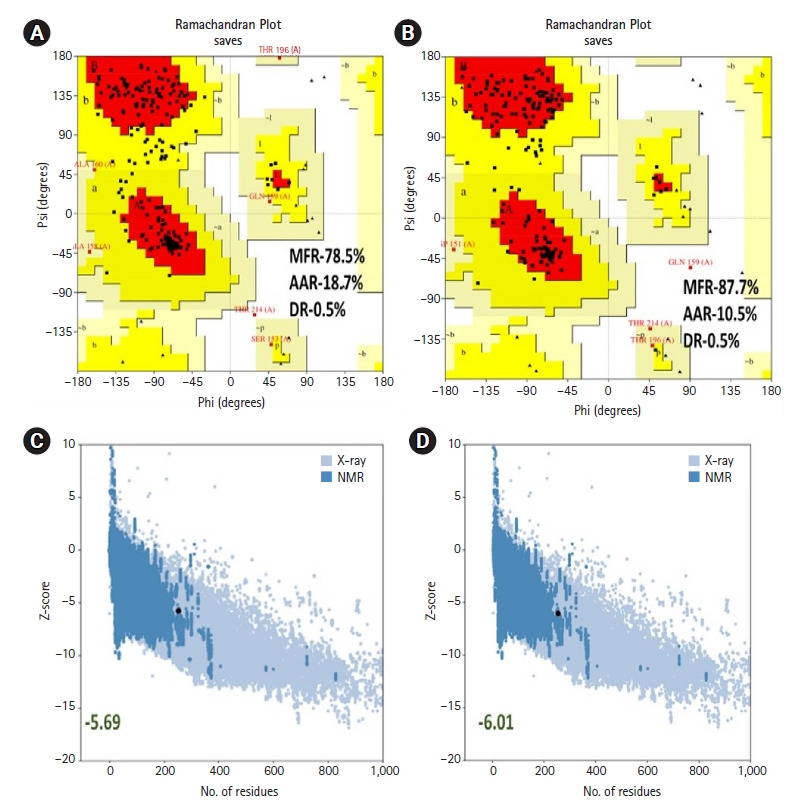
(A, B) Analysis of Ramachandran plot PROCHECK server. The MFR, AAR, GAR, and DR was represented the most favored, additional allowed, generously allowed, and disallowed regions of vaccine. (C, D) 3D structure validation with a Z-score by Pro-SA server.

**Fig. 7.**
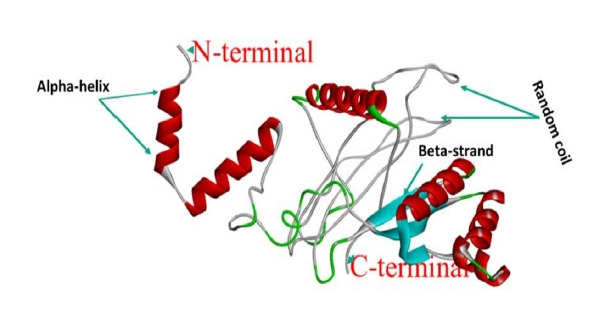
The tertiary structure of the designed vaccine construct.

The RaptorX server was utilized as the best template to build the top five models in homology modeling. We chose the model with the lowest C-score (–4.87), as advised by the server, out of the five. With GDT-HA score 0.8176, RMSD value 0.519, MolProbity 2.993, Clash score 25.7, and Poor rotamers score 0.8, the vaccine (model 1) exhibited 87.7% residues in the favorable area in the Ramachandran plot after refinement. The ProSA-web servers were used to further evaluate the refined 3D vaccine model. The vaccine's Ramachandran plot showed 78.5% residues in the favorable zone, 18.7% in approved regions, and 0.5% residues in prohibited regions before refining. The Ramachandran plot of the refined vaccine model showed 87.7% residues in the favorable region and 10.5% in allowed regions, while 0.5% residues in disallowed regions (Fig. 6B). Similarly, the crude model had a Z-score of –5.69, but the refined model had a Z-score of –6.01 (Fig. 6D). Fig. 7 shows a structural depiction of the developed vaccine.

### Molecular docking studies

**Fig. 8. f8-gi-21065:**
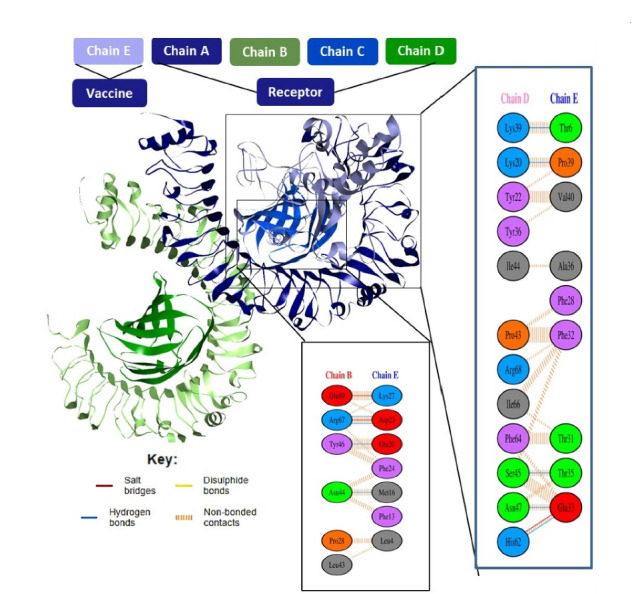
Molecular docking between the vaccine and the TLR4 receptor. TLR4, Toll-like receptor 4.

To predict their binding affinity and interactions, the vaccine (ligand) and TLR4 (receptor) were docked. As a result, the ClusPro v2.0 server produced ten docked complexes in various positions. We chose the complex with the lowest energy score and the binding posture with functional interactions from among them. As a result, model 1 met the inclination criterion. As a result, it was chosen as the best vaccine–TLR4 complex, with a –937.6 energy score. Binding interactions and residues implicated in active site residues were investigated in the chosen complex. A total of eight hydrogen bonds were found in the interaction surface. There were eight classical hydrogen bonds among the hydrogen bonds. The interacting residues in the CHB from the vaccine were Lys39, Lys20, Ser45, Asn47, His62, Arg67, and Asn44. Moreover, associated TLR4 active site residues are shown in Fig. 8. Other hydrogen bond interactions were as follows: three were electrostatic salt bridges, zero were disulphide bonds and nine single non-banded contact.

### Molecular dynamics simulation

**Fig. 9.**
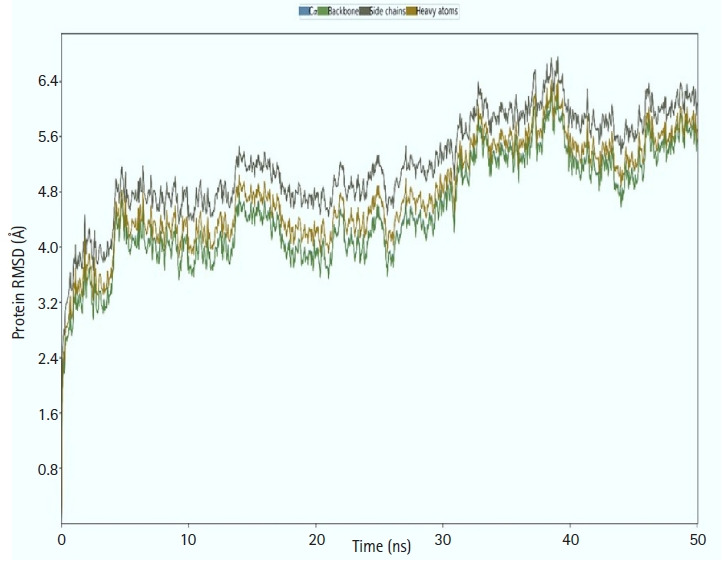
Molecular dynamic simulation of the multi-epitope vaccine complex. The root mean square deviation (RMSD) plot of the backbone atoms of the complexes.

**Fig. 10.**
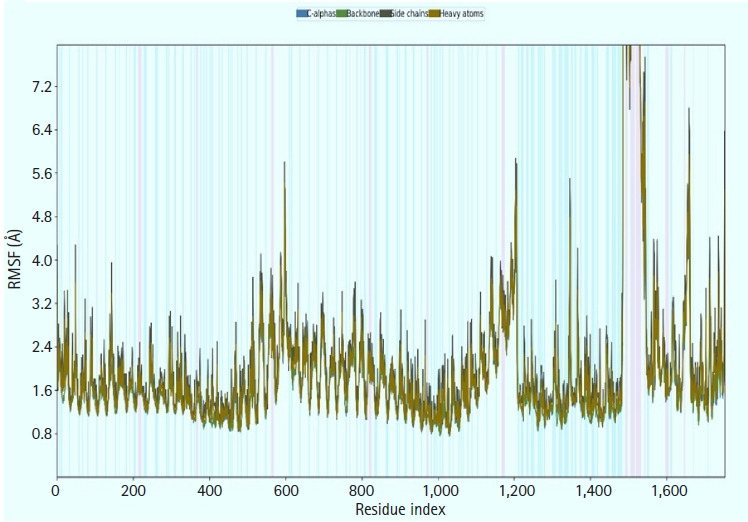
Molecular dynamic simulation of the multi-epitope vaccine complex. The root mean square fluctuation (RMSF) plot of the multi-epitope docked vaccine candidate. α-helical and β-strand regions are highlighted in red and blue backgrounds, respectively. These regions are defined by helices or strands that persist over 70% of the entire simulation.

We calculated the RMSD for the vaccine complex and the vaccine. The vaccine complex had an average RMSD of 4.76Å, which indicated structural stability during the interaction. Fig. 9 shows that the vaccination complex has an early rise in RMSD characteristics until 5 ns, following which it becomes stable until 15 ns. From 15 to 25 ns, there was a decreased degree of fluctuation, which might be responsible for structural integrity and/or allowing solid binding. Moreover, the protein flexibility across the amino acid residues was evaluated through the RMSF score. The RMSF profile of the vaccine complex indicates maximum amino acid residues from complexes that an RMSF profile below 4.0 Å and greater change was observed for fewer residues. This result from Fig. 10 defines the vaccine complex stability and stiffness.

### Immune response simulation

**Fig. 11.**
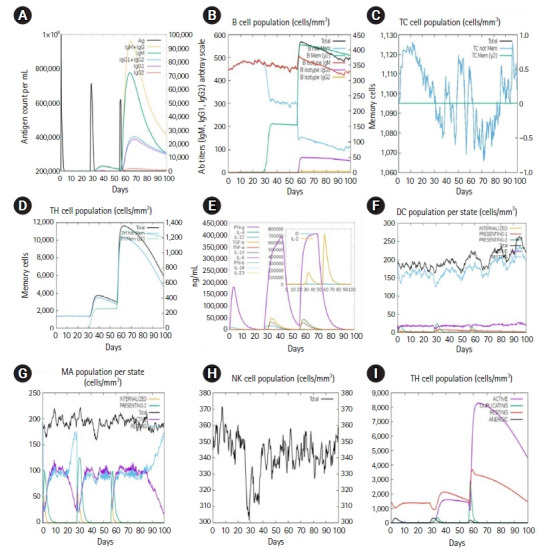
Immune response triggered by the designed vaccine. The graph shows primary, secondary and tertiary immune responses (A), B-cell population (B), cytotoxic T-cell population (C), helper T-cell population (D), induction of cytokines and interleukins (E), dendritic cell population per state (F), macrophage (MA) population per state (G), natural killer (NK) cells (total count) (H), and percentage (%), and amount (cells/mm^3^) of Th1 mediated (I).

The simulated immune response in Fig. 11 mimicked the immune response induced by certain infections. Secondary and tertiary immune responses, for instance, were greater than primary immunological responses (Fig. 11A). Secondary and tertiary responses revealed larger levels of antibodies (IgG1 + IgG2, IgM, and IgG + IgM), which correlated with an antigen extenuation showing the establishment of memory cells, resulting in increased antigen clearance after subsequent exposures (Fig. 11A). Furthermore, B-cells, cytotoxic T cells, and helper T cells had a longer time of survival, indicating class flipping between immune cells and IgM memory development (Fig. 11B–11D). The Th0 type immune reaction had a lower proportion (%) and number (cells/mm^3^) than the Th1 type immune reaction (Fig. 11I). Expanded macrophage mobility was seen during the presentation, but dendritic cell movement was predicted (Fig. 11F and 11G).

### Codon adaptation and *in-silico* cloning

**Fig. 12.**
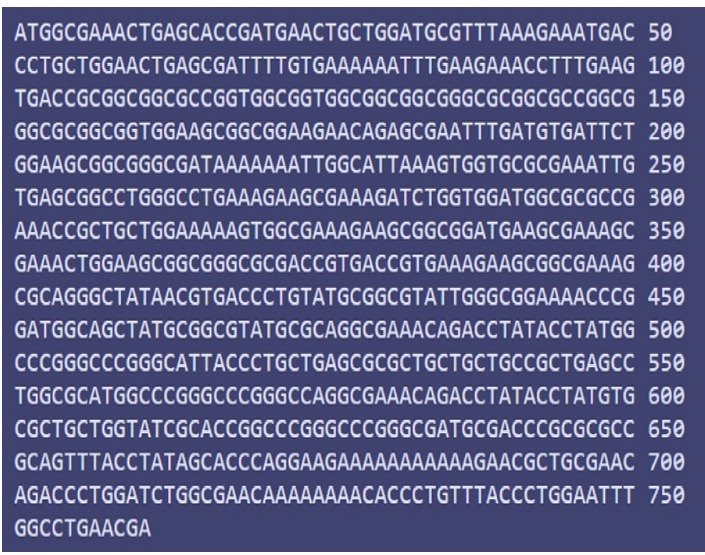
Codon adaptation of Epstein-Barr virus to *Escherichia coli* K12 strain.

**Fig. 13.**
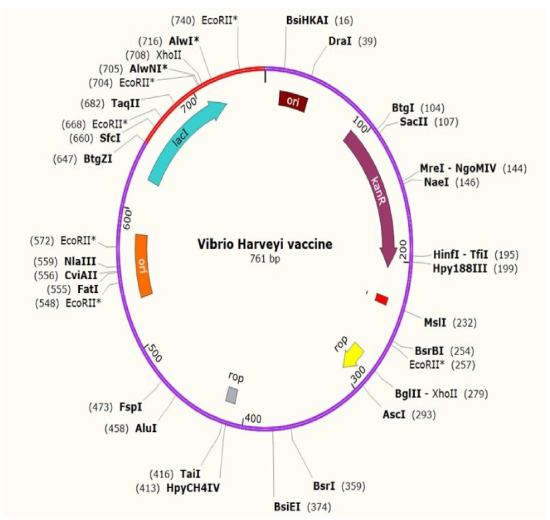
The proposed vaccine was cloned into the pET-28a (+) vector *in silico*.

To improve the translation efficiency of the vaccine design, we adjusted the codons according to the *E. coli* K12 on the JCat service. The nucleotide sequences created by the peptide vaccine construct (254 amino acid residues) totaled 761 lengths (Fig. 12). Furthermore, the modified nucleotide sequence has a GC content of 59.39% and a CAI value of 0.62, respectively. We used *Xho*I and *Bam*HI restriction sites as the start and end cut points, accordingly, to insert the modified sequence into the pET28a (+) vector. Using the SnapGene program, the optimized vaccine design was cloned into the pET28a (+) cloning vector (Fig. 13).

## Discussion

The current diabolical emergence of *Vibrio harveyi* causing vibriosis poses a serious danger to the worldwide aquaculture industry [72], which influences us to use an immunoinformatics method to build this multi-epitope vaccination. The vaccination based on the hemolysin protein displayed outstanding relevance as predicted by immunoinformatics, proving our effort to be reliable. A vaccine protects against infectious illnesses in a safe and effective manner [73]. Acquired immunity against contagious diseases should be possible with it [74]. As a result of this study, we designed a vaccine based on epitopes that would provide a strong immune response to *V. harveyi*. *V. harveyi* infection and transmission are difficult to control and prevent in the absence of an effective vaccine. Furthermore, in order to regulate the current situation, effective immunization has yet to be produced. As a result, a novel vaccine development strategy is critical to finding a solution to the current economically threatening aquaculture problem. Because the hemolysin of *V. harveyi* is important for immunological invasion and fish-to-fish transmission, our goal was to develop an epitope vaccination that targeted the hemolysin. In order to enable cellular and humoral immune systems to recognize this protein, the hemolysin protein surface was evaluated for its antigenic region. Previously, scientists develop an *in-silico* designing of epitope-based vaccine against the seven banded grouper nervous necrosis virus affecting fish species [31]. As vibriosis is very common in tilapia species and in the past studies MHC class I and class II molecules were found in the experimental data of cord and tilapia for starting immune responses against infections. So, this multi-epitope vaccine targeting HLA-A*0201, HLA-B*3501, and HLA-B*3508 might be an efficient vaccination against certain fish diseases [21,23]. The first step was identifying all possible CTL, HTL, and LBL epitopes. Next, vaccines were designed with three antigenic epitopes—CTL, HTL, and LBL—since the linkers below corresponded to the top three epitopes. They were used in vaccine development as an important component that improves the stability, folding, and transcriptional regulation of our peptide vaccine [75]. The adjuvant was attached to the CTL epitope by EAAAK linker, which helps to induce high levels of both cellular and immunogenic humoral responses for particular antigens, and amplify the vaccine’s stability and longevity [76]. A total of 254 amino acid residues were found in the vaccine construction. An essential characteristic of a recombinant vaccine is its solubility, a type of physicochemical property [77]. A solubility assessing tool was used to determine whether the vaccine construct was solvable inside the host *E. coli*, and the results showed that it was solvable. The vaccine's nature, as indicated by the theoretical PI value, was acidic. The protein's stability index, as recommended by server tools, indicates that it will be stable following synthesis. The GRAVY (grand average of hydropathicity index) value and aliphatic index, on the other hand, indicated that the vaccine was hydrophilic and thermostable, respectively. According to the prediction of physicochemical properties and scores on all parameters, there is a high probability for this vaccine to be a valid candidate against hemolysin protein of *V. harveyi*. The detected models were revised and the best model (based on the lowest energy score) was chosen after the 3D structure prediction (based on C-score). We observed a reasonable number of Z-score (–6.01) and superior features of most favored, acceptable, and prohibited areas for the Ramachandran plot in the validation test of 3D structure. It was suggested by the lowest energy score of 937.6 for a molecular docking between the peptide vaccine and virus glycoprotein binding convenient receptor of TLR4 that the vaccine could have infection-inhibiting activity and might interact tightly with TLR4 receptor. The molecular dynamics simulation is a potentially useful tool for understanding how proteins function and how their structure is derived. Anatomical movement can be simulated by protein dynamic simulations as a function of time. We have performed dynamic simulations of the vaccine candidate for 50 ns, and analyzed the results using the RMSD and RMSF scores. When comparing distinct atomic conformations of a molecular system, the RMSD value is employed. A significant flexibility and departure of vaccine candidates from receptor structure was determined using the RMSD value, whereas the displacement of our particular vaccine candidate's atoms from receptor structure was determined using RMSF of the complex structure. The calculated average RMSD and RMSF value was 4.77 Å and 4.0 Å, respectively. The fluctuation was not observed to be larger in the vaccine section, but it smoothed out after 5 ns, suggesting that the modeled vaccine and receptor are stable. Lastly, we examined the optimal target clearance and cell density parameters for the best immunologic response against the pathogen by constructing an immune simulation. As a result of the upgraded vaccine doses, the immune system created memory B-cells (with a half-life of several months) and T cells. The vaccination efficiently imitated a humoral immune response to increased immunoglobulin production in this way. In order to optimize the multi-epitope vaccine production, the MD simulation was done to evaluate stability of the vaccine candidate with the receptor, in which codon optimization was done for stability of the construct vaccine within the host. Eventually, the codon was adjusted, and *in-silico* cloning of the intended vaccine candidate into the *E. coli* K12 expression host pET28a (+) vector was successful.

A range of computational techniques were used in this work to find possible T- and B-cell epitopes in *V. harveyi* hemolysin protein, which were finally stitched into a multi-epitope mRNA vaccine. The newly developed vaccine possesses the immunodominant qualities that are sought. Significantly, it was capable of binding to the immunological receptor TLR4 and induce a substantial immune response in regard to *V. harveyi* infection. Based on our findings, we believe that developing a vaccine against the etiological agent of the *V. harveyi* outbreak in fish should begin with the vaccine candidate. In addition, the possible epitopes discovered in this study can be employed in future research. Nevertheless, more testing is needed to show that our designed vaccine is an effective preventive against *V. harveyi* infection in fish species.[Fig f5-gi-21065][Fig f8-gi-21065]

## Figures and Tables

**Table 1.** The selected CTL epitopes for the final vaccine construction

Epitope	C-score	Antigenicity	Immunogenicity	Toxicity	Allergenicity
AQGYNVTLY	1.0098	1.5077	Positive	Negative	Negative
WAENPDGSY	1.8227	0.6526	Positive	Negative	Negative
AQAKQTYTY	1.2614	1.0896	Positive	Negative	Negative

CTL, cytotoxic T lymphocyte.

**Table 2.** The selected HTL epitopes for the final vaccine construction

Epitope	Antigenicity	IFN-γ	IL-4	IL-10	Toxicity	Allergenicity
ITLLSALLLPLSLAH	0.6989	Positive	Inducer	Inducer	Negative	Negative
QAKQTYTYVRCWYRT	0.4670	Positive	Inducer	Inducer	Negative	Negative
DATRAPQFTYSTQEE	0.8194	Positive	Inducer	Inducer	Negative	Negative

HTL, helper T-lymphocyte; IFN-γ, interferon γ; IL, interleukin.

**Table 3.** The selected LBL epitopes for the final vaccine construction

Epitope	Probability	Antigenicity	Allergenicity	Toxicity
KERCEQTLDLAN	0.327	0.8769	Negative	Negative
NTLFTLEFGLND	0.405	0.8716	Negative	Negative

LBL, linear B-lymphocyte.

**Table 4.** Binding affinities and interaction between selected epitopes and HLA alleles

T-cell epitope	HLA allele	Epitope affinity (kcal/mol)	Control affinity (kcal/mol)	No. of hydrogens bonds (CHB)	Residues involved in CHB networks (n)
AQGYNVTLY	HLA-B*3508	‒7.2	‒9.2	7 (6)	Gln69, Trp149, Thr7, Ile8, Met19, Ala2, Ile7, (7)
WAENPDGSY	HLA-A*0201	‒6.1	‒8.2	7 (5)	Tyr84, Lys146, Val2, Thr7, Val9, Asn77, Thr143 (7)
AQAKQTYTY	HLA-B*3501	‒8.4	‒8.2	9 (8)	Tyr9, Leu8, Thr7, Glu166, Lys66, Arg170, Tyr4, Trp167, Ala1 (9)
ITLLSALLLPLSLAH	DRB1*07:01	‒5.9	‒6.9	9 (7)	Arg71, Thr77, Asn82, Ala12, Thr13, Val14, Val1, Glu6, Ser4 (9)
QAKQTYTYVRCWYRT	DRB1*04:01	‒6.1	‒6.7	12 (10)	Tyr7, Asp9, Asp9, Ser24, Glu63, Lys66, Arg69, Arg69, Tyr99, Glu152, Glu152, Gln155 (12)
DATRAPQFTYSTQEE	DRB1*11:01	‒6.8	‒7.3	9 (7)	Asp29, Lys58, Thr8, Asp30, Thr233, Ser57, Gln5, Glu212, Lys4 (9)

HLA, human leukocyte antigen.

**Table 5.** Antigenic, allergenic and physicochemical characteristics of the construct

Characteristic	Finding	Remark
No. of amino acids	254	Suitable
Molecular weight	27,044.64	Average
Theoretical pI	4.95	Acidic
Chemical formula	C_1214_H_1914_N_310_O_379_S_4_	-
Extinction coefficient (at 280 nm in H_2_O)	27515	-
Estimated half-life (mammalian reticulocytes, *in vitro*)	30 h	-
Estimated half-life (yeast-cells, *in vivo*)	>20 h	-
Estimated half-life (*Escherichia coli, in vivo*)	>10 h	-
Instability index of vaccine	20.25	Stable
Aliphatic index of vaccine	82.05	Thermostable
Grand average of hydropathicity (GRAVY)	‒0.237	Hydrophobic
Antigenicity	0.7017	Antigenic
Immunogenicity	0.7122	Immunogenic
Allergenicity	No	Non-allergen
Solubility	0.891723	Soluble

**Table 6.** The secondary structural features of the vaccine construct

Feature	SOPMA server		PSIPRED server	
	Amino acid	Percentage	Amino acid	Percentage
Alpha helix	84	33.07	109	42.91
Beta strand	43	16.93	52	20.47
Random coil	127	50	93	36.61

SOPMA, Self-Optimized Prediction Method with Alignment; PSIPRED, PSI-blast based secondary structure prediction.
